# Removing barriers to advanced imaging and machine learning-based analysis

**DOI:** 10.1242/jcs.264705

**Published:** 2026-08-12

**Authors:** Jodie R Malcolm, Stuart Lacy, Andrea Papaleo, Le Liu, Suleiman H Kwairanga, Graeme Park, Joanne Marrison, Olympia Physouni, Richard Kasprowicz, Kerry Hallbrook, Richard Williams, Kildare Miranda, Teng-Leong Chew, Caron A Jacobs, Mahmoud Bukar Maina, Ana Paula C A Lima, Jeremy C Mottram, Beth A Cimini, Ben Powell, Peter O’Toole, Laura Wiggins, William J Brackenbury

**Affiliations:** 1 Jack Birch Cancer Research Unit, Department of Biology, University of York, Heslington, York, UK; 2 York Biomedical Research Institute, Department of Biology, University of York, Heslington, York, UK; 3 IT Services, University of York, Heslington, York, UK; 4 Imaging Platform, Broad Institute of MIT and Harvard, Cambridge, MA, USA; 5 Biomedical Science Research and Training Centre, Yobe State University, Damaturu, Nigeria; 6 Sussex Neuroscience, School of Life Sciences, University of Sussex, Brighton, UK; 7 Bioscience Technology Facility, Department of Biology, University of York, Heslington, York, YO10 5DD, UK; 8 GlaxoSmithKline Medicines Research Centre, Stevenage, UK; 9 UK Cell Culture and Banking, Discovery Sciences, AstraZeneca, Cambridge, UK; 10 ioLight, Whitchurch, Hampshire, UK; 11 Instituto de Biofisica Carlos Chagas Filho, Universidade Federal do Rio de Janeiro, Rio de Janeiro, Brazil; 12 Advanced Imaging Center and Integrative Imaging, Howard Hughes Medical Institute Janelia Research Campus, Ashburn, VA, USA; 13 Department of Pathology and Institute of Infectious Disease and Molecular Medicine, University of Cape Town, Cape Town, South Africa; 14 Department of Mathematics, University of York, Heslington, York, UK; 15 School of Chemical, Materials and Biological Engineering, University of Sheffield, Sheffield, UK

## Abstract

Global and community-driven initiatives have recently achieved considerable success in overcoming key challenges that hinder the widespread adoption of advanced microscopy and bioimage analysis tools in under-resourced settings. To build upon this progress, we held a workshop in May 2025 at the University of York, UK to address the needs and barriers associated with implementing time-lapse imaging and machine learning-based phenotyping in low-resource research environments. We focussed on identifying the specific challenges faced by the existing networks represented at the meeting, emphasising how integrating combined imaging hardware and machine learning-based approaches can solve these problems. This article summarises the key observations and actionable strategies made at the workshop. These proposed steps aim to significantly increase the dissemination and uptake of these powerful technologies to advance biological research in low-resource settings globally.

## Introduction

Significant progress has been made in advancing imaging technologies to enable extraction of high-quality information from light microscopy images, thus enabling in-depth characterisation of processes spanning areas of research including tissue biology, immunology, microbiology and oncology. Moreover, by adding repeated measurements of live cell cultures to generate time-lapse imaging data, it is also possible to gain insight into dynamic changes in cellular behaviour over time. However, such measurements typically rely on data obtained using high-cost systems, including advanced confocal, super-resolution or ptychographic microscopy - a technique that computationally reconstructs high-resolution images from overlapping illumination diffraction patterns. In addition, emerging machine learning-based image analysis tools and approaches often require specialist expertise and significant computational resources, the latter of which depends on stable and fast access to the cloud and/or costly hardware.

The Technopolis Group is an international research and consulting organisation working in the public sector throughout Europe, Africa, Latin America and the Caribbean. The purpose of the Technopolis Group is to collect evidence and scientific insight to provide strategic advice and operational support to decision makers with emphasis on understanding societal challenges and shaping a sustainable society. In their recent survey of barriers affecting progress in bioimaging, they identified high costs of imaging equipment and limited access to analysis software as major hurdles impeding the progress of bioimaging research in low- and middle-income countries (LMICs) (Davé *et al*., 2023). The survey also highlighted that, despite resource constraints, data interoperability is essential to enable researchers in LMICs to extract and analyse relevant information (Davé *et al*., 2023). The proliferation of an array of lower-cost and open-source light microscopes presents an opportunity to increase uptake of such imaging techniques and image analysis technologies by researchers in LMICs. For example, a brand-new confocal microscope can easily cost upwards of $300,000, with higher specification instruments dramatically exceeding this price, whereas open-source community-designed microscopes can cost as little as $1.35, with more specialised instruments that are 3D-printed and customisable averaging ~$350. However, these more frugal systems also present challenges, particularly in relation to data quality and imaging modality. In particular, lower-cost imaging systems are often limited to brightfield acquisition and are more susceptible to artefacts. Further challenges include creating awareness, improving access, incentivising continued use, addressing local scientific needs, and encouraging sharing and investment, discussed extensively elsewhere (Aaron *et al*., 2025).

To increase dissemination and uptake of advanced light microscopy and image analysis technologies, it is necessary to develop tools and workflows that can extract relevant information without the need for high-cost imaging equipment or high-performance computing hardware. The first step in such workflows is acquisition, where a balance needs to be struck between frugality and minimum requirements for production of research-level datasets (Aaron *et al*., 2025). However, it is important to recognise that research in low-resource settings is not just limited by access and that it generates biological and social insights that cannot be generated elsewhere in the world. LMIC-led studies address region-specific diseases, agricultural challenges and genetic diversity that are globally under-represented. Strengthening local imaging capacity expands the scientific landscape, enabling scientific discoveries that benefit the entire field. In this context, emerging approaches such as U-Nets – a type of convolutional neural network (CNN) – generative adversarial networks (GANs) and diffusion models, which can enable label-free organelle prediction using data acquired on high-end systems, need to be adapted so they can be used with data from low-cost systems. Doing so is not only a matter of accessibility but also a scientific necessity – models trained on globally diverse, locally generated datasets are more robust, more generalisable and more representative of real biological variation.

In May 2025, we held a workshop at the University of York to discuss needs and barriers to time-lapse imaging and machine learning-based phenotyping in low-resource settings. The meeting focused on identifying the challenges faced via existing networks, including the African Bioimaging Consortium (ABIC), the Biomedical Science Research and Training Centre (BioRTC), the Africa Microscopy Initiative (AMI) and Latin America Bioimaging (LABI). We examined how combined imaging hardware and machine learning-based approaches - i.e., solutions where image analysis software is designed to work on, or with, associated hardware - can address these challenges. This Perspective article summarises key observations from the workshop and the steps we identified that can be taken to increase dissemination and uptake of these technologies.

## Barriers to advanced imaging and image analysis in low-resource settings

A range of interconnected barriers contribute to the inequitable adoption of advanced imaging and analysis among researchers in LMICs (Marey *et al*., 2026). To address this inequity, our recent discussions identified key challenges that disproportionately affect researchers from such backgrounds. We hope these insights will inform more inclusive strategies for the propagation of advanced microscopy technologies and machine learning-based bioimage analysis tools.

### Education and training

Education and training opportunities are paramount for mastering standard laboratory techniques and for developing and maintaining expertise in image acquisition and downstream analysis. However, building the capacity for such opportunities is impeded by significant barriers that necessitate prompt action to propel locally-directed research output (De Niz *et al*., 2024).

Mentorship is vital for meaningful scientific progress. By encouraging junior researchers to take ownership of their ideas, learning and development, guided by an experienced mentor, knowledge transfer becomes intuitive and instinctive. This dynamic not only advances education and training but also promotes a culture of investing in the success of future researchers. Structured mentoring schemes are uncommon in low resource settings and are often considered inadequate when measured against high-income country (HIC) standards. Most mentoring guidelines are developed with the assumption of abundant resources, which makes their uptake in low-resource settings particularly challenging (Chi *et al*., 2019). Tailored “mentoring-the-mentor” or “training-the-trainer” initiatives specifically developed within the context and culture of LMICs have been designed to spearhead investment and implementation of mentoring practices within institutional settings (Lescano *et al*., 2019).

In addition to low adoption of mentoring programmes, there is a general lack of access to education and training opportunities for researchers in LMICs, which further contributes to poor dissemination of knowledge on bioimaging techniques. Conferences, workshops and teaching events often do not reach communities that are suffering from infrastructure constraints. At such events, opportunities for training on imaging technologies or fostering bioimage analysis knowledge exchange are disproportionately over-represented by attendees from HICs *versus* LMICs (Velin *et al*., 2021).

Although bioimaging hubs including the BioRTC exist to overcome these training inadequacies, researchers often need travel bursaries to access such facilities. Global inequality in funding opportunities and grant success in favour of HICs further disadvantages researchers in resource-constrained settings seeking opportunity for professional development (Charani *et al*., 2022). Training in grant writing and research management is also not widely available across LMIC institutions, impacting the competitiveness of applications. The AMI is pioneering change in access to microcopy training and networking opportunities. By delivering all-expenses-paid microscopy and analysis courses along with themed workshops to tackle locally-relevant health challenges, the AMI is an exemplar for promoting microscopy where it is not widely used.

### Lack of technical specialists

A consequence of the limited education and training opportunities in bioimage analysis for LMICs described above is a shortage of technical specialists in core facilities or research institutes. Technical specialists are crucial for sustaining and developing bioimage analysis capabilities, with key roles including managing day-to-day operations of the research facility, maintaining the microscopes(s) and providing training on microscope usage, data collection, analysis and interpretation. Given the high turnover of research staff and students in many institutions, technical specialists are therefore essential for retaining institutional knowledge of bioimage practices. In particular, trained bioimage analysts preserve expertise in advanced microscopy, manage large-scale data storage and regularly use complex image analysis tools. However, fewer than 25% of these critically-needed specialists work outside of Europe or North America (Corbat *et al*., 2025). Low retention rates of technical specialists in LMICs further exacerbates this issue. The so-called “brain-drain” of trained researchers migrating from LMICs to HICs highlights the challenges that scientists face in resource-constrained settings when it comes to building research capacity and accessing bioimaging for their research. The resulting lack of relevant technical expertise in LMICs can perpetuate poor uptake and use of advanced microscopy and analysis in core facilities and research institutes and might impede important discoveries essential to progressing region-specific research.

Compounding these issues is the often limited technical support offered by international suppliers of microscopy platforms and image analysis tools, a challenge further intensified in LMIC institutions where constrained resources reduce the capacity to trial new technologies or sustain pilot initiatives. In these settings, the barrier is often not a lack of institutional willingness but a lack of financial and infrastructural ability to invest in, maintain and grow new ventures, making the absence of robust external technical support an even more significant bottleneck. Furthermore, researchers in HICs frequently benefit from around-the-clock assistance if an instrument breaks down or requires specialist support, whereas those in LMICs can experience significant delays and cost before a service engineer can attend their site. These delays can be further impeded by poor internet connection and time zone differences, which hinder the effectiveness of remote troubleshooting and support. In addition, the pace at which microscope parts can be imported to replace broken components provides a further bottleneck. Even if cutting-edge microscopes are donated or purchased by researchers in LMICs, the lack of access to local service engineers and spare parts means these instruments risk being underutilised and creates a dependency on external collaborators for access to advanced imaging opportunities (Davé *et al*., 2023).

Taken together, inadequate access to bioimage-centred education and training in LMICs prohibits access to and retention of essential technical experts, who are fundamental to sustaining institutional knowledge of microscopy technologies and analytical tools. Restricted access to manufacturers’ technical support presents a further issue for researchers in low-resource settings. Such challenges risk making advanced microscopy and bioimage analysis less attractive approaches for scientists looking to address key research questions in their local communities.

### Insufficient physical and technical infrastructure

There are considerable physical and technical infrastructure challenges for the adoption of advanced microscopy and bioimage analysis in LMICs. Here, we define physical infrastructure to include tangible, material resources, such as an operable research laboratory and essential microscopy equipment hardware, whereas technical infrastructure encompasses the digital tools and computing resources that are essential for storing and processing large bioimage datasets. Key considerations for implementing responsible, LMIC-feasible machine learning workflows in bioimage analysis are outlined in Box 1.

In terms of physical infrastructure, LMICs experience inequitable access to affordable and reliable electricity, with the majority of the most disadvantaged in this regard residing in sub-Saharan Africa. These challenges arise as a result of systemic issues, including a lack of electricity-use frameworks, unreliable electrical supply, a lack of financial investment and infrastructure inadequacies (Brew-Hammond, 2010). Invariably, these issues result in disproportionate access to electrical supply and frequent power outages across the African continent. To a lesser extent, electricity generation and distribution is also an ongoing challenge for other LMICs, including across Latin America and Caribbean (LAC) countries. For many LACs, more than 70% of electricity is generated using renewable sources, with hydropower contributing substantially to this distribution. However, despite significant economic growth over the last decade and substantial uptake of renewable energy sources to accommodate electrical requirements, many LACs still lack a reliable and renewable electricity supply (González-Lorente *et al*., 2024).

Poor electrical infrastructure distribution has significant consequences for growth and economic potential of LMICs and also adds additional constraints to research facilities that require a reliable electricity supply for the operation of advanced microscopes and for culturing live cells, as well as for maintaining cold storage temperatures for research samples and consumables. A reliable supply of electricity is of particular importance for time-series experiments using live cells and/or tissues. With respect to microscopy hardware and image analysis solutions, this issue can be overcome through the use of uninterruptible power supplies (UPS) and/or power banks located within frugal microscopes. In addition, data storage and use of high performance computing (HPC) for data processing is one of the most energy-intensive scientific endeavours, which also relies on provision of a reliable electricity supply. To overcome these limitations, diesel generators are often used to accommodate electrical requirements in low-resource settings. However, the use of generators has environmental and economic implications. The actual costs of these generators vary widely depending on the local price of fuel; however, they are estimated to range from three to seven times the cost of purchasing electricity directly from the grid (Farquharson *et al*., 2018).

Advanced microscopy systems, such as confocal, super-resolution and quantitative phase imaging (QPI) microscopes, can exceed hundreds of thousands of dollars to purchase. For many LMIC institutions these costs are prohibitive, creating a barrier to bioimaging research. Initiatives such as the AMI aim to address this gap in technical infrastructure by establishing shared imaging hubs to provide equitable access to high-end instruments and training (Reiche *et al*., 2023). Complementary strategies exist, such as the OpenFlexure project, OpenFrame platform and Simplifying Quantitative Imaging Platform Development and Deployment (Squid) platform, all of which offer customisable microscopes that can be locally manufactured using 3D-printing and open-source designs at a fraction of commercial costs (Li *et al*., 2020; Lightley *et al*., 2023; Knapper *et al*., 2024; Malcolm *et al*., 2026). Crucially, these affordable systems have the capacity to address pressing research questions in health and disease (Knapper *et al*., 2024). However, frugal microscopes can be perceived as being “not research grade”, limiting their integration into research workflows. Validation studies and community-driven standards for these accessible platforms are needed to shift this perception and promote their adoption in resource-limited settings (Aaron *et al*., 2025).

Given that modern bioimage analysis workflows are often computationally exhaustive, an additional consideration is limited network bandwidth, intermittent internet connectivity and reliance on low-cost computers or mobile phones to access the web (Nacis *et al*., 2024). This severely restricts the use of advanced bioimaging analysis pipelines, constraining research progress. Furthermore, in our experience, high-resolution QPI modalities such as the PhaseFocus LiveCyte or the Tomocube HT-X1 can easily generate large-scale datasets ranging from hundreds of gigabytes to several terabytes in size. Storing and processing these data requires graphics processing units (GPUs), HPC clusters and/or reliable cloud access, which are often not readily accessible in LMIC research environments. Although cloud-based data processing solutions exist, their adoption is constrained by connectivity issues and cost barriers to sustained use (Davé *et al*., 2023). This lack of affordable computing infrastructure further compounds the challenge of storing and processing large datasets generated by high-end microscopes.

### Other considerations

Additional barriers to advanced microscopy and image analysis workflows were discussed extensively during our workshop. High-quality sample preparation is fundamental for advanced imaging, yet researchers in LMICs frequently face barriers in accessing essential reagents and consumables, such as antibodies, foetal bovine serum (FBS) and trypsin, owing to restrictive import policies, high costs and long delivery times. These constraints can significantly delay experiments and limit reproducibility, underscoring the need for regional reagent hubs and open-source protocols to mitigate supply chain vulnerabilities.

Crucially, the increasing cost of accessing and publishing in research journals deprives skilled researchers in resource-constrained settings. Although there has been a positive trend in institutional open access (OA) publishing since 2008, disparities remain. Researchers in LMICs encounter disproportionally higher costs for publishing primary research in OA journals or face limited global reach for their work under current publishing policies (Kilgallon *et al*., 2023).

Furthermore, collaborative research is a cornerstone of modern science, but institutional policies can sometimes create disincentives. In some LMIC research environments, promotion and performance evaluation systems prioritise first or corresponding authorship, reducing the perceived value of multi-author publications. This can discourage participation in large, collaborative projects that are essential for capacity building and global integration. Reforming academic reward systems to recognise diverse contributions, such as data sharing, method development and training, would help foster more equitable collaborations.

**Fig. 1:**
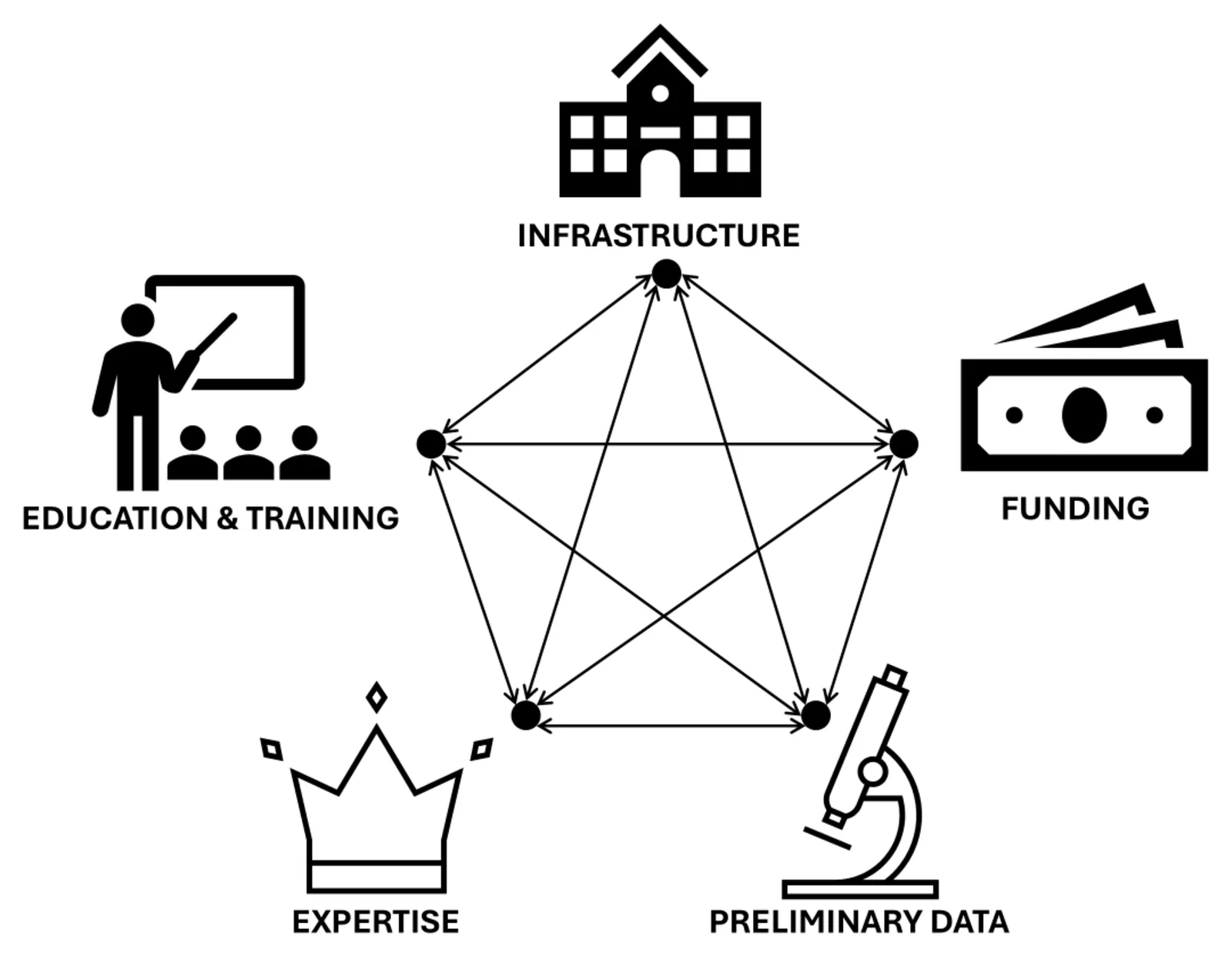
Barriers to advanced imaging and analysis are interconnected and compound one another. Key challenges, including limited education and training, inadequate infrastructure and a shortage of technical expertise, impede the generation of preliminary data, which is crucial for securing funding from competitive research grants. These issues do not exist in isolation; instead, they are interconnected obstacles that collectively hinder the production of high-quality research focused on locally relevant health concerns. The absence of preliminary data perpetuates economic constraints, which in turn restrict the development of training programs, infrastructure and local technical capacity.

In summary, there are multiple local and systemic challenges faced by researchers in low-resource settings when implementing advanced microscopy and bioimage analysis workflows to tackle urgent regional health challenges. These issues stretch beyond funding disparities and include limited training opportunities in bioimage analysis, inadequate physical and technical infrastructure and institutional governance that may not encourage collaboration and knowledge sharing. Importantly, these are not stand-alone issues with straightforward solutions – they are deeply interconnected, forming a vicious cycle (Fig. 1). Researchers in resource-constrained settings often lack the preliminary data needed to submit competitive grant applications, yet without funding they cannot generate the data required to secure that funding. This “catch-22” perpetuates inequality and hinders progress in building sustainable research capacity in bioimage analysis.

## Progress and future directions

During our meeting, we reflected on how achieving widespread access to advanced imaging and analysis in low-resource settings relies on progress in three interconnected areas: strong networks and capacity-building programmes that support training, collaboration and sustainability; the development of low-cost, accessible hardware; and development of image analysis software with machine learning capabilities that can run on modest computing infrastructure. Progress in each of these areas is already underway and here we highlight examples that reflect advances in practice, ranging from innovative hardware and software solutions through to community-driven initiatives.

### Geographical and organisational structures enable success

Successful collaboration, particularly in the global south, depends on overcoming administrative and infrastructure barriers, such as customs restrictions, visa limitations and low data bandwidth, as well as improving access to appropriate low-cost, low maintenance instrumentation. This is particularly the case for travelling researchers and expert technologists, as well as movement of samples and, to a lesser extent, data. Adopting a hub-and-node model with a central, well-supported coordinating hub may facilitate formation of networks that connect regional centres of excellence across the global south with wider, local nodes that are using more affordable imaging platforms (De Niz *et al*., 2024).

There are numerous examples of existing collaborative programmes that actively support bioimaging research in LMICs (Table 1). These initiatives play a crucial role in reducing barriers to training, equipment access and community participation. A notable example is the Imaging4All (I4A) programme funded by the Wellcome Trust, which provides travel support for researchers in LMICs to attend specialist bioimaging courses and gain hands-on access to cutting-edge microscopy facilities (https://globalbioimaging.org/i4a/news/i4a). By enabling researchers to train directly on advanced instruments and engage with international experts, programmes like I4A help to build sustainable local capacity and strengthen global bioimaging networks. When considering development of new networks and/or extension of existing programmes, it is essential to complement these partnerships and avoid redundancy by pursuing opportunities to enhance existing initiatives and promote interoperability with ongoing projects. It is also necessary to set appropriate participation criteria and accountability frameworks that ensure resources are used transparently and effectively.

Opportunities to engage with industry should also be sought during network development, particularly given the rapid pace at which microscopy technologies are being developed, deployed and deprecated. Industry partners, including large pharmaceutical companies offer crucial bioimaging research assets such as advanced instruments, high-value datasets and specialist expertise. However, these organisations may be hesitant to share such resources due to concerns around intellectual property, competitive advantage or regulatory constraints. To overcome this barrier, bioimaging networks need to develop mutually beneficial arrangements that clearly outline shared value, whether through collaborative research, training exchanges or co-development of tools. Also important is the creation of robust ethical frameworks that enable responsible equipment and data access as well as data (re)use. Such frameworks will help to build trust, reduce perceived risks and enable industry participation in ways that strengthen the wider bioimaging community.

Finally, it is essential to balance short-term research goals with potential for long-term impact, such that initial phases of collaborations prioritise generating pilot data to support future investment while longer-term strategies focus on ensuring sustainability of new technologies and their uptake beyond the initial funding cycle. A key element of this prioritisation is embedding frequent horizon scanning - systematically monitoring emerging technologies, threats to current technologies, developing methods and sector-wide shifts - in project planning, to enable research agility through flexible, modular workflows that support innovation and local responsiveness to changing research priorities.

### Enabling knowledge sharing and synergy

Effective training is essential for the widespread adoption and longevity of advanced microscopy and bioimage analysis in low-resource settings, and excellent progress has been made (Table 1). Future programmes should aim to integrate mentorship schemes into in-person workshops and ensure accessibility for users who have varying levels of experience with complex bioimage analysis workflows. Beyond instrument operation, training should encompass experimental design and optimisation, piloting and troubleshooting imaging workflows, data interpretation, grant writing and research management. Ongoing successful initiatives like AMI and ABIC and low-cost platforms like OpenFlexure can serve as practical resources for hands-on learning and local innovation. With respect to skilled technical staff, training programmes should not only include facility maintenance, software troubleshooting, pipeline resilience, and introduction to coding and machine learning methods, but also consider how to deliver effective training to imaging facility users and the next generation of bioimage analysts.

There also exists significant opportunity for bioimage analysis to be embedded within university curriculum, particularly through the integration of frugal open-hardware instruments, such as the OpenFlexure Microscope, alongside lightweight, open-source analysis tools. Bringing these resources into undergraduate and postgraduate training would help to cultivate microscopy-proficient technical specialists much earlier in their careers. Equally important is the expansion of interdisciplinary and collaborative training within institutions, so that biomedical scientists, engineers and computer scientists can learn and problem-solve together. Strengthening these interdepartmental connections would foster the collaborative environment needed to accelerate hardware and software co-development and to support the next wave of technical specialists who will drive innovation in bioimaging.

Clear communication around realistic timelines for research outputs, expectations of deliverables, and upfront acknowledgement of resource constraints and limitations is paramount to maintain trust among researchers. Open and transparent planning with stakeholders will help to align expectations between local bioimage scientists, international collaborators and funders, and will ensure that bioimaging projects remain achievable and deliver significant impact in local communities. Networks should be built around shared scientific priorities – e.g., infectious diseases, oncology and neurobiology – as this will foster interdisciplinary collaboration and accelerate knowledge transfer. Linking projects around mutual research interests could also enable resource pooling and the development of standardised workflows, which are essential for reproducibility and scalability. In addition, global initiatives should respect and integrate regional research agendas, ensuring that projects address locally relevant scientific and medical needs. Encouraging local teams to define their own priorities not only enhances the relevance of research but also strengthens long-term sustainability and community ownership.

### Enabling access to hardware

The hub-and-node network model can help enable access to microscopes, our discussion highlighted that it does not solve all access issues. Service contracts for maintaining microscopes are generally unaffordable in anything but the short term. Sourcing replacement parts is also very difficult and import times for replacement parts can become a significant rate limiting step to advance bioimaging research; at the same time, it is impractical to keep duplicate spare parts in each localised country. Even in HICs, companies are running leaner centralised stocks to decrease waste and costs. Although not a replacement for cutting-edge high-end microscopes, portable microscopes could be one enabling solution, especially for the collection of preliminary data. Prices for portable microscopes vary significantly depending on customisable features such as resolution, magnification, illumination type and digital imaging capability (Table 2). Another solution is to make software tools and workflows hardware-agnostic and adaptable to both high-end and resource-constrained environments, with expectations set accordingly. There also needs to be a focus on producing tools that have acceptable performance on lower-end devices but that will also benefit from higher computational resources if and when they are available.

More advanced microscopy features enhance performance but will likely also increase cost. Thus, a minimal setup needs to be balanced against the level of information required for meaningful biological inference from microscopy images. Determining this balance is crucial for designing affordable, purpose-fit tools for research, diagnostics or education in resource-limited settings. Care is also needed to ensure that the image quality is sufficient not to frustrate the analysis and thus the end user, which can have a negative effect of discouraging microscopy use rather than encouraging growth in this area. However, access to low-cost microscopes is of utmost importance to allow researchers to optimise protocols and develop initial datasets that can then help them access better equipped hubs. This approach would be akin to that used for structural 3D electron microscopy, in which most central hubs require users to have tested their samples on a more basic local system before being given precious access time on a heavily used centralised microscope.

### Enabling access to reagents (or finding ways to not require them)

Our discussion on the key challenges facing LMIC researchers in adopting advanced bioimaging tools also revealed that limited access to reagents remains a major bottleneck. Regional procurement strategies that can benefit different communities of researchers need to be explored. However, for fundamental research, much can still be gained through label-free imaging (Suman *et al*., 2016), which decreases the reliance on high-cost reagents and reduces long lead times associated with importing, optimising and validating labeled antibodies. Moreover, by simply enabling robust, quantitative time-lapse imaging, significant experimental results can be obtained, especially with the latest developments in software analysis (Wiggins *et al*., 2023). Furthermore, advances in techniques such as live cell painting (the use of a membrane-permeable metachromatic dye that enables dual colour phenotypic live-cell staining without the requirement for additional stains) and stain prediction (a computational approach that uses deep learning algorithms to digitally “stain” live cells without using physical fluorescent markers) could also serve to reduce the need for costly bioimaging reagents (Cross-Zamirski *et al*., 2022, 2023; Garcia-Fossa *et al*., 2025).

### Enabling access to software advances

To enable its widespread uptake by researchers in LMICs, microscopy analysis software needs to strike a balance between analytical capability and computational efficiency. Advanced functionalities, such as deep learning capabilities, multi-dimensional visualisation or single-cell and time-series feature extraction, can offer deeper insights, but they often require access to HPC resources that may not be available in resource-limited settings. Furthermore, to directly address locally relevant research questions posed by LMIC researchers, tools that allow users to retrain image analysis models or adjust parameters are essential to enable their adaptation to diverse sample types and imaging conditions. Designing software that remains responsive, adaptable and enables biologically meaningful interpretation of results, whilst also being able to run efficiently on modest hardware, is crucial for supporting impactful and accessible research in low-resource environments. See Table 3 for some examples.

## Broadening microscopy and imaging analysis technology adoption

Numerous other papers have eloquently discussed potential solutions to increase uptake of microscopy and imaging analysis technologies in resource-constrained settings (Reiche *et al*., 2023; Rahmoon *et al*., 2024; De Niz *et al*., 2024; Bajcsy *et al*., 2025; Aaron *et al*., 2025; Antunes *et al*., 2025). With respect to the adoption of bioimage analysis technology, data pipelines, which cover the pathway from hypothesis to publication, integrating quality control, analysi and reproducibility at each step, should be implemented (Schmied *et al*., 2024). Minimum standards for participation (e.g., staffing, connectivity, equipment) should be defined to ensure equity and efficacy. Furthermore, new infrastructures are needed to support secure, scalable and flexible data storage. Although cloud solutions are attractive when local hardware is limited, they face technical and legal limitations (with respect to data crossing borders) and can be cost-prohibitive, as well as trust challenges related to data ownership and sovereignty. Such challenges with on-site infrastructure (e.g. power and networking reliability) can make cloud solutions attractive and hybrid approaches, such as the Segment Anything demo application, are expected to dominate (Ouyang *et al*., 2023).

The software landscape is changing quickly, and those working on smaller, less demanding software are now in the minority. This skills gap for software is analogous to that for hardware and includes those working to produce modular, customisable (and repairable) hardware and software combinations. Closing this gap will require an emphasis on developing hardware solutions and analytical models that require less computational power than the current state-of-the-art. Finally, personalised and automated guidance systems (e.g., AI tools, tutorials, peer help desks) will help make such advanced analysis accessible. Successful examples include: the https://forum.image.sc/forum (Rueden *et al*., 2019), Global BioImage Analysts’ Society (GloBIAS) help sessions (https://www.globias.org/activities/globias-free-help) and the Bioimage.IO chatbot (Lei *et al*., 2024), as well as a growing array of open source educational tools, such as Microtutor (https://microtutorcourses.org/), and the Bioimaging Guide (Senft *et al*., 2023).

In conclusion, we have outlined some key systemic challenges that were discussed in our recent workshop and that hinder the widespread adoption of advanced microscopy and bioimage analysis tools in under-resourced settings. We have also highlighted a superb array of global and community-driven initiatives working to overcome these disparities, with considerable success. However, there is a need for coordinated strategies that go beyond infrastructure and funding. To foster meaningful uptake of these emerging technologies, we need to prioritise knowledge sharing, capacity building and equitable collaboration. By aligning training, communication and research priorities with local contexts, the global bioimaging community can create a collaborative ecosystem that drives innovation where it is needed most.

## Figures and Tables

**Table 1:** Community-driven initiatives to support bioimaging research in LMICs

Initiative	Description	Benefit to LMICs	Website
**African Bioimaging Consortium (ABIC)**	A network of imaging scientists and life science researchers with an interest in and use for microscopy throughout Africa. ABIC aims to unite the African microscopy community and support the development of bioimaging across the continent. It is supported by the Chan Zuckerberg Initiative (CZI) “Expanding Global Access to Bioimaging” program.	ABIC provides a mechanism for sharing and accessing information, ideas and expertise. Additionally, members collaborate to build community-based solutions to key challenges and coordinate to engage with vendors, policy makers and global partners.	https://www.africanbioimaging.org/
**Africa Microscopy Initiative (AMI)**	A multi-faceted program focused on increasing access to and expertise in bioimaging throughout Africa. It achieves this through the open access AMI Imaging Centre, which offers African scientists access to advanced microscopy equipment and support on an application basis, along with various training and teaching programs including workshops and webinars. AMI also facilitates the equipment distribution Program for Equipment Exchange & Reutilization (PEER), which partners with commercial organisations to distribute pre-owned, high-quality microscopes to institutions across the continent.	Equipment and expertise are shared in tandem across a community of researchers with similar priorities.	https://www.microscopy.africa/
**Biomedical Science Research and Training Centre (BioRTC)**	A regional bioimaging hub based in Yobe State University, Nigeria. Its goal is to offer state-of-the-art core facilities and training to biomedical science researchers within and outside Nigeria, as well as foster research collaborations.	The facility contains one of the largest concentrations of high-end microscopy equipment in west Africa. The centre actively promotes open access to bioimaging technologies, provides structured training for early-career researchers and creates opportunities for hands-on capacity building.	https://biortc.com/
**Global Bioimaging**	A community network for all stakeholders and participants in bioimaging, funded by the European Union’s Horizon 2020 programme. Particular expertise in training and knowledge transfer is delivered via webinars and job-shadowing or placements. Their Imaging for All (i4A) programme funds researchers in LMICs to visit well-resourced bioimaging facilities. To make this programme operate successfully, they have developed an application process for vetting, coordinating and financing visits. Global Bioimaging also delivers “train-the-trainer” courses designed for imaging core facility professionals on how to organise and deliver effective training programs, perpetuating knowledge sharing and bioimaging community engagement.	The network provides a route to scaling up research projects, providing access to resources at one scale that are used to generate data needed to justify resources at the next scale.	https://globalbioimaging.org/
**Imaging Africa**	Hosts pan-continental, fully-funded workshops to build microscopy expertise among African researchers, covering costs include meals, air transport and accommodation to ensure workshops are fully accessible. This enables participants from all African nations to gain hands-on training in advanced imaging techniques and strengthen the regional imaging community.	Imaging Africa directly addresses barriers faced by researchers in LMICs such as lack of training opportunities and limited funding by providing free, high-quality microscopy education.	https://www.training.microscopy.africa/imaging-africa
**Latin America Bioimaging (LABI)**	A network of bioimaging scientists from the Latin America and Caribbean region. LABI focuses on training, education and providing researchers in the region with access to appropriate imaging technologies.	LABI aims to strengthen and promote access to bioimaging technologies via a collaborative network encompassing training, knowledge exchange and infrastructure access.	https://labi.lat/
**OpenScopes Initiative**	Coordinated from Imperial College London (with partners in India, China and South Africa and sponsored in part by the Biotechnology and Biological Sciences Research Council (BBSRC), Engineering and Physical Sciences Research Council (EPSRC), Medical Research Council (MRC), Research England, CZI and the Wellcome Trust), the initiative encourages the use of flexible, modular hardware and open source software for instrument control and image analysis.	The initiative opens a path for researchers in LMICs that has minimal reliance on the most expensive proprietary microscopy products.	https://openscopes.com/
**South Africa Bioimaging (SABI)**	A national network that connects imaging scientists across South Africa to share expertise, support new labs and promote best practices in microscopy. It provides access to imaging facilities and staff contacts across provinces, facilitates training opportunities, and offers protocols for image acquisition, processing and analysis. SABI also helps researchers source key materials like fluorescent probes and integrates the South African imaging community into the broader global bioimaging network.	SABI strengthens microscopy capacity within South Africa by promoting resource sharing, standardisation and skills development. Its open-access model, training support and integration with global networks help overcome common barriers in LMICs such as limited infrastructure, fragmented expertise and difficulty accessing reagents or technical guidance.	https://www.sabioimaging.org/
**SWIFT Awards**	Mobility grants, facilitated by ABIC, that support African researchers in gaining access to advanced microscopy infrastructure, training and international collaboration opportunities. These awards are designed to make it easier for researchers in Africa to attend workshops, use imaging facilities or engage with global microscopy networks.	SWIFT Awards reduce the financial and logistical barriers for researchers in LMICs by covering the costs associated with travel and equipment access. This supports the development of a more equitable and connected bioimaging research community in low-resource settings.	https://www.africanbioimaging.org/swift-awards
**West African Microscopy and Bio-Imaging Analysis Network (WAMBIAN)**	A network of scientists and researchers in West Africa that promote microscopy and bioimage analysis techniques within the region, provide training and support, and promoting collaboration and resource sharing among members.	WAMBIAN supports LMICs by building regional expertise in microscopy, reducing the reliance on external training and equipment. It enables institutions to share knowledge, infrastructure and resources to strengthen the overall scientific ecosystem in West Africa.	https://www.wambian.org/

**Table 2:** Recent advances in low-cost imaging hardware

Microscope	Description	Benefit to LMICs	Cost	References
**CellScope**	CellScope is a mobile microscopy platform that transforms a smartphone into a diagnostic tool using optical attachments. Its use in diagnostics has already been exemplified for malaria, tuberculosis, cervical cancer and ear infections.	1-5 µm resolution for standard versions; advanced prototypes with higher-end optics can go below 1 µm. CellScope clips onto a smartphone and provides 20x-100x magnification, ideal for disease detection and research in LMICs where access to lab infrastructure is limited. It digitally stores and shares diagnostic images, allowing data collection for research, monitoring and public health decision-making, even in areas without internet connectivity, with images synced to the cloud once a connection becomes available.	A research-grade mobile microscope typically costs £4-£49, with clinical-grade smartphone attachments typically costing around £237.	(Skandarajah *et al.*, 2014)
**Foldscope**	Foldscope is an ultra-low-cost light microscope made from a lens, magnets and paper. Its lightweight and compact design makes it ideal for fieldwork, classrooms and educational outreach.	2 µm resolution and magnification up to 140x, suitable to observe bacteria, cells and other key biological samples. Made primarily from a single sheet of waterproof paper folded into a microscope structure, making it incredibly lightweight and compact. Uses ambient or phone-based light sources with no need for batteries, power outlets or lab infrastructure.	Typically costs £1 - £10 per unit.	(Cybulski *et al.*, 2014)
**Glowscope**	The Glowscope is a DIY fluorescence microscope that transforms a smartphone or tablet into a functional microscope using simple, off-the-shelf parts.	Offers around 10 µm resolution with ~5x magnification, sufficient to visualise fluorescent tissues and embryos, useful in basic biomedical research and training.	£24–£39 per unit.	(Schaefer *et al.*, 2023)
**ioLight**	The ioLight microscope is a portable, high-resolution digital microscope designed for use in the field or in the lab where space and mobility are key. The microscope offers time-lapse imaging capabilities for observing dynamic processes.	1 µm resolution and up to 400x magnification, sufficient to visualise cellular structures. Works on battery, displays images directly on a smartphone or tablet, and does not require internet access. Designed for mobile use in veterinary clinics, agricultural fields, environmental surveys and incubators.	£2,200 - £6,600 per unit.	https://iolight.co.uk/
**Matchboxscope**	The Matchboxscope, also known as the ESPressoscope platform, is an open-source, low-cost, and highly portable brightfield microscope designed for capturing micrographs in the field and within cell incubators.	Resolution between 3-4 µm and supports z-stacking for 3D reconstruction. Full schematics, software and assembly instructions are freely available, encouraging local manufacturing, customisation and repair. Compact, lightweight, and ideal for remote or rural use, supporting field diagnostics and research without the need for lab infrastructure or continuous internet connectivity.	Typically costs £25 - £65.	(Li *et al.*, 2024)
**Octopi**	Octopi is a low-cost, open-source, automated slide-scanning microscope designed for clinical diagnostics and pathology. It features motorised scanning, autofocus and digital imaging, supporting both brightfield and fluorescence modes.	Octopi uses a Raspberry Pi camera or similar CMOS sensor with a high-numerical-aperture lens and 10x or 20x objectives, delivering ~1.3–2.0 μm resolution, comparable to a basic lab microscope and suitable for clinical screening and AI-based analysis of stained samples. It has low power consumption, with some versions running on battery packs or solar systems, and fully open hardware and software, with 3D-printable parts and readily available components, encouraging local assembly, customisation and repair.	£195 - £390 per unit.	(Li *et al.*, 2019)
**OpenFlexure**	The OpenFlexure microscope is a low-cost, open-source and 3D-printable digital microscope designed to provide lab-grade performance with high customisability. The microscope supports motorised sample positioning and focus control, enabling automation for scanning large areas and time-lapse imaging.	Resolution below 500 nm, with highly customisable components such as objective lenses, cameras and illumination to suit a range of different sample types. Core components can be 3D-printed and designs are open-source. This enables local manufacturing, reducing reliance on expensive imports and lengthy supply chains. OpenFlexure is fully functional offline with no internet or cloud dependency.	£160 for a complete kit of parts needed to assemble the microscope, or £200-£300 for pre-assembled versions.	(Collins *et al.*, 2020)

**Table 3:** Examples of advances in accessible image analysis software

Software	Description	Benefit to LMICs	References
**CellPose**	Software built around a neural network with exceptional image segmentation abilities.	CellPose software is highly effective, with some graphical user interface (GUI) accessibility. The code base could enable expert software engineers in LMICs to repurpose code for their own use cases.	(Stringer *et al.*, 2021)
**CellProfiler** (Carpenter *et al.*, 2006)	Open-source tools for developing image analysis pipelines with application to high throughput data.	Pipeline-oriented analyses are highly customisable for diverse use cases and allow for computation to be scheduled or managed off-site.	(Carpenter *et al.*, 2006)
**DeepCell Kiosk**	A sophisticated computation management system for faster, more efficient image processing.	LMIC labs can efficiently share computational resources (specifically, but not exclusively, GPU chips) hosted at a small number of hub institutions.	(Bannon *et al.*, 2021)
**Ilastik**	A suite of machine learning algorithms with simple GUIs that enable non-experts to train them.	This project accounts for potential coding skills gaps between software creators and users.	(Berg *et al.*, 2019)
**ImageJ/Fiji**	Robust, versatile open-source software with a long history of collaborative development.	The long history and large community of users mean that there is greater chance that users can find advice and assistance with their analyses.	(Schneider *et al.*, 2012)
**Napari**	Image analysis software with particular application to high-dimensional data (e.g. 3 spatial dimensions and/or time).	In combination with appropriate hardware, researchers in LMICs can explore image features that are unavailable in 2D snapshots.	(Sofroniew *et al.*, 2026)
**Piximi**	A browser/cloud-based platform for image analysis and annotation.	Piximi is user friendly and has functionality for both cloud-based and local computation. This may be particularly useful for compromises that need to be made in terms of local computational resources and data security and sensitivity issues.	(Moser *et al.*, 2024)
**ZeroCostDL4Mic**	A curated library of customisable jupyter notebooks for a variety of bioimaging tasks. By default, computation is provided by free Google servers.	These resources are useful both for performing analyses and for training software engineers in LMICs.	(von Chamier *et al.*, 2021)

## Summary statement

This Perspective article conveys insights from a 2025 workshop on advancing timelapse imaging and machine learning-based phenotyping, outlining practical strategies to expand access to cutting-edge bioimaging in low-resource settings.

## Box 1 LMIC-feasible considerations for responsible machine learning-based image analysis

Importance of data quality controls

Data that are collected for the purpose of training machine learning-based image analysis tools needs to incorporate strict quality controls (QC). Without accurate data, machine learning models can learn an incorrect representation of the input. High-quality data allows for accurate extraction of information across diverse imaging settings and improved statistical inference. To ensure that imaging data are of high quality - particularly in areas where imaging conditions, hardware availability and environmental factors may vary widely - simple, standardised QC procedures are critical for ensuring datasets remain usable across diverse contexts. These should include illumination checks, focus metrics and rigorous metadata capture. QC pipelines should be adopted early in the experimental design processes to ensure that the data are of high quality.

Need for validation

Models that have been trained in one setting rarely generalise perfectly to another. Therefore, validation is an important step in machine learning-based image analysis and should include cross-site testing, hold-out datasets i.e., portions of data withheld from the model training and used only for validation and testing to assess model performance on previously unseen examples, and simple performance metrics that can be conducted without specialist infrastructure. For LMICs, transparent error reporting, model interpretability and human-in-the-loop review will ensure that outputs remain trustworthy and robust, even when computational resources are limited.

Reproducibility and maintenance

Reproducibility relies on clear documentation, version control and lightweight deployment formats that can run on modest hardware. Maintenance should prioritise models that can be retrained incrementally as new data becomes available and workflows that depend on open-source, community-driven tools to avoid vendor lock-in – i.e., dependence on proprietary platforms or container technologies which make it difficult to migrate pipelines elsewhere. Containerised environments (e.g. Docker), which package code and dependencies into portable, reproducible units, can help to ensure long-term reproducibility even in resource-constrained settings, and reduce the risk of container lock-in.

Data ownership and governance

Ethical and sustainable machine learning practice requires locally governed data policies that respect institutional autonomy and national regulations. LMIC institutions should retain ownership of imaging data and define clear agreements for data sharing, reuse and model training. Governance frameworks should emphasise FAIR principles (findability, accessibility, interoperability and reusability), transparent consent and protection of sensitive biological or clinical information, aligning with global initiatives such as the Global Bioimaging Data Policy Framework (Winter *et al*., 2026).

Training and oversight

Responsible deployment of machine learning requires ongoing training for local researchers, technicians and students, focusing on practical skills such as annotation, QC, model evaluation and troubleshooting. Oversight mechanisms, such as peer review of annotations, periodic model audits and community-based troubleshooting forums will help ensure that machine learning tools remain accurate and ethically deployed. Embedding these practices within local curricula and research networks will strengthen long-term capacity.

## References

[R1] Aaron JS, Jacobs CA, Malacrida L, Keppler A, French P, Fletcher DA, Wood C, Brown CM, Wright GD, Ogawa S, Maina M (2025). Challenges of microscopy technology dissemination to resource-constrained communities. Nat Methods.

[R2] Antunes MM, Oliveira AG, de Paula CM, Chew T-L, Paula-Neto HA, Menezes GB (2025). Bioimaging Brasil: democratizing in vivo optical microscopy to drive scientific progress across a vast nation. Nat Methods.

[R3] Bajcsy P (2025). Enabling global image data sharing in the life sciences. Nat Methods.

[R4] Bannon D, Moen E, Schwartz M, Borba E, Kudo T, Greenwald N, Vijayakumar V, Chang B, Pao E, Osterman E, Graf W (2021). DeepCell Kiosk: scaling deep learning-enabled cellular image analysis with Kubernetes. Nat Methods.

[R5] Berg S, Kutra D, Kroeger T, Straehle CN, Kausler BX, Haubold C, Schiegg M, Ales J, Beier T, Rudy M, Eren K (2019). Ilastik: Interactive machine learning for (bio)image analysis. Nat Methods.

[R6] Brew-Hammond A (2010). Energy access in Africa: Challenges ahead. Energy Policy.

[R7] Carpenter AE, Jones TR, Lamprecht MR, Clarke C, Kang IH, Friman O, Guertin DA, Chang JH, Lindquist RA, Moffat J, Golland P (2006). CellProfiler: image analysis software for identifying and quantifying cell phenotypes. Genome Biol.

[R8] von Chamier L (2021). Democratising deep learning for microscopy with ZeroCostDL4Mic. Nat Commun.

[R9] Charani E, Abimbola S, Pai M, Adeyi O, Mendelson M, Laxminarayan R, Rasheed MA (2022). Funders: The missing link in equitable global health research?. PLOS Glob Public Health.

[R10] Chi BH, Belizan JM, Blas MM, Chuang A, Wilson MD, Chibwesha CJ, Farquhar C, Cohen CR, Raj T (2019). Evaluating academic mentorship programs in low- and middle-income country institutions: Proposed framework and metrics. Am J Trop Med Hyg.

[R11] Collins JT, Knapper J, Stirling J, Mduda J, Mkindi C, Mayagaya V, Mwakajinga GA, Nyakyi PT, Sanga VL, Carbery D, White L (2020). Robotic microscopy for everyone: the OpenFlexure microscope. Biomed Opt Express.

[R12] Corbat AA, Walther CG, de la Ballina LR, Condon ND, Felder AA, Schätz M, Schmerl B, Sugawara K, Prats C, Klemm A, Levet F (2025). GloBIAS: strengthening the foundations of BioImage Analysis. ArXiv.

[R13] Cross-Zamirski JO, Anand P, Williams G, Mouchet E, Wang Y, Schönlieb C-B (2023). Class-guided image-to-image diffusion: Cell painting from Brightfield images with class labels. arXiv [csCV].

[R14] Cross-Zamirski JO, Mouchet E, Williams G, Schönlieb C-B, Turkki R, Wang Y (2022). Label-free prediction of cell painting from brightfield images. Sci Rep.

[R15] Cybulski JS, Clements J, Prakash M (2014). Foldscope: origami-based paper microscope. PLoS One.

[R16] Davé A, Varnai P, Babb R, Raabe B, Amato M (2023). Landscape review of barriers affecting progress in the field of Bioimaging.

[R17] De Niz M (2024). Building momentum through networks: Bioimaging across the Americas. J Microsc.

[R18] Farquharson D, Jaramillo P, Samaras C (2018). Sustainability implications of electricity outages in sub-Saharan Africa. Nat Sustain.

[R19] Garcia-Fossa F, Moraes-Lacerda T, Rodrigues-da-Silva M, Diaz-Rohrer B, Singh S, Carpenter AE, Cimini BA, de Jesus MB (2025). Live-cell painting: Image-based profiling in live cells using acridine orange. Mol Biol Cell.

[R20] González-Lorente Á, Hernández-López M, Martín-Álvarez FJ, Mendoza-Jiménez J (2024). A comparative analysis of electricity generation in Latin America and the Caribbean using multivariate techniques. Heliyon.

[R21] Kilgallon JL, Khanna S, Dey T, Smith TR, Ranganathan K (2023). Open(ing) access: Top health publication availability to researchers in low- and middle-income countries. Ann Glob Health.

[R22] Knapper J, Whiteford F, Rosen D, Wadsworth W, Stirling J, Mkindi C, Mduda J, Sanga VL, Nyakyi PT, Mboa Nkoudou TH, Jafsia E (2024). Developing the OpenFlexure Microscope towards medical use: technical and social challenges of developing globally accessible hardware for healthcare. Philos Trans A Math Phys Eng Sci.

[R23] Lei W, Fuster-Barceló C, Reder G, Muñoz-Barrutia A, Ouyang W (2024). BioImage.IO Chatbot: a community-driven AI assistant for integrative computational bioimaging. Nat Methods.

[R24] Lescano AG, Cohen CR, Raj T, Rispel L, Garcia PJ, Zunt JR, Hamer DH, Heimburger DC, Chi BH, Ko AI, Bukusi EA (2019). Strengthening mentoring in low- and middle-income countries to advance global health research: An overview. Am J Trop Med Hyg.

[R25] Li E, Saggiomo V, Ouyang W, Prakash M, Diederich B (2024). ESPressoscope: A small and powerful approach for in situ microscopy. PLoS One.

[R26] Lightley J, Kumar S, Lim MQ, Garcia E, Görlitz F, Alexandrov Y, Parrado T, Hollick C, Steele E, Roßmann K, Graham J (2023). openFrame: A modular, sustainable, open microscopy platform with single-shot, dual-axis optical autofocus module providing high precision and long range of operation. J Microsc.

[R27] Li H, Krishnamurthy D, Li E, Vyas P, Akireddy N, Chai C, Prakash M (2020). Squid: Simplifying quantitative imaging platform development and deployment. bioRxiv.

[R28] Li H, Soto-Montoya H, Voisin M, Valenzuela LF, Prakash M (2019). Octopi: Open configurable high-throughput imaging platform for infectious disease diagnosis in the field. bioRxiv.

[R29] Malcolm JR, Physouni O, Lacy S, Bentley M, Howarth SP, Macdonald S, Droop AP, Powell B, Wiggins L, Brackenbury WJ, O’toole PJ (2026). Adapting the OpenFlexure microscope for affordable live-cell imaging. bioRxiv.

[R30] Marey A, Ambrozaite O, Afifi A, Agarwal R, Chellappa R, Adeleke S, Umair M (2026). A perspective on AI implementation in medical imaging in LMICs: challenges, priorities, and strategies. Eur Radiol.

[R31] Moser LM, Gogoberidze N, Papaleo A, Lucas A, Dao D, Friedrich CA, Paavolainen L, Molnar C, Stirling DR, Hung J, Wang R (2024). Piximi - An Images to Discovery web tool for bioimages and beyond. bioRxivorg.

[R32] Nacis JS (2024). Barriers and enablers to the effective implementation of omics research in low- and middle-income countries. Nat Biotechnol.

[R33] Ouyang W, Eliceiri KW, Cimini BA (2023). Moving beyond the desktop: prospects for practical bioimage analysis via the web. Front Bioinform.

[R34] Rahmoon MA, Hobson CM, Aaron JS, Balasubramanian H, Chew T-L (2024). More than just “added value”: The perils of not establishing shared core facilities in resource-constrained communities. J Microsc.

[R35] Reiche MA, Jacobs CA, Aaron JS, Mizrahi V, Warner DF, Chew T-L (2023). A comprehensive strategy to strengthen bioimaging in Africa through the Africa Microscopy Initiative. Nat Cell Biol.

[R36] Rueden CT, Ackerman J, Arena ET, Eglinger J, Cimini BA, Goodman A, Carpenter AE, Eliceiri KW (2019). Scientific Community Image Forum: A discussion forum for scientific image software. PLoS Biol.

[R37] Schaefer MA, Nelson HN, Butrum JL, Gronseth JR, Hines JH (2023). A low-cost smartphone fluorescence microscope for research, life science education, and STEM outreach. Sci Rep.

[R38] Schmied C (2024). Community-developed checklists for publishing images and image analyses. Nat Methods.

[R39] Schneider CA, Rasband WS, Eliceiri KW (2012). NIH Image to ImageJ: 25 years of image analysis. Nat Methods.

[R40] Senft RA, Diaz-Rohrer B, Colarusso P, Swift L, Jamali N, Jambor H, Pengo T, Brideau C, Llopis PM, Uhlmann V, Kirk J (2023). A biologist’s guide to planning and performing quantitative bioimaging experiments. PLoS Biol.

[R41] Skandarajah A, Reber CD, Switz NA, Fletcher DA (2014). Quantitative imaging with a mobile phone microscope. PLoS One.

[R42] Sofroniew N (2025). napari: a multi-dimensional image viewer for Python. Zenodo.

[R43] Sofroniew N (2026). napari: a multi-dimensional image viewer for Python. Zenodo.

[R44] Stringer C, Wang T, Michaelos M, Pachitariu M (2021). Cellpose: a generalist algorithm for cellular segmentation. Nat Methods.

[R45] Suman R, Smith G, Hazel KE, Kasprowicz R, Coles M, O’Toole P, Chawla S (2016). Label-free imaging to study phenotypic behavioural traits of cells in complex co-cultures. Sci Rep.

[R46] Velin L, Lartigue J-W, Johnson SA, Zorigtbaatar A, Kanmounye US, Truche P, Joseph MN (2021). Conference equity in global health: a systematic review of factors impacting LMIC representation at global health conferences. BMJ Glob Health.

[R47] Wiggins L, Lord A, Murphy KL, Lacy SE, O’Toole PJ, Brackenbury WJ, Wilson J (2023). The CellPhe toolkit for cell phenotyping using time-lapse imaging and pattern recognition. Nat Commun.

[R48] Winter SL, Moore J, Tavares AAS, Galloway G, Dojat M, Longo DL, Sullivan R, Mathur A, Chaabane L (2026). A global effort toward standards for data sharing in biomedical imaging : Developing Consensus and Infrastructure for Global Data Interoperability: Developing Consensus and Infrastructure for Global Data Interoperability. EMBO Rep.

